# Active Learning and Technology Approaches for Teaching Immunology to Undergraduate Students

**DOI:** 10.3389/fpubh.2020.00114

**Published:** 2020-05-07

**Authors:** Sharon A. Stranford, Judith A. Owen, Frances Mercer, Roberta R. Pollock

**Affiliations:** ^1^Biology Department, Pomona College, Claremont, CA, United States; ^2^Department of Biology, Haverford College, Haverford, PA, United States; ^3^Department of Biological Sciences, California State Polytechnic University, Pomona, CA, United States; ^4^Department of Biology, Occidental College, Los Angeles, CA, United States

**Keywords:** active learning, concept maps, immunology education, just-in-time teaching, student-centered learning, technology in education, undergraduate

## Abstract

Immunology is a fascinating and extremely complex field, with natural connections to many disciplines both within STEM and beyond. Teaching an undergraduate course in immunology therefore provides both opportunities and challenges. Significant challenges to student learning include mastering the volume of new vocabulary and figuring out how to think coherently about a physiological system that is so anatomically disseminated. More importantly, teaching immunology can be complicated because it requires students to integrate knowledge derived from prior introductory courses in a range of fields, including cell biology, biochemistry, anatomy and genetics. However, this also provides an opportunity to use the study of the immune system as a platform on which students can assemble and integrate foundational STEM knowledge, while also learning about a new and exciting field. Pedagogical theory has taught us that students learn best by engaging with complicated questions and by thinking metacognitively about how to approach solutions. Building this skill set in today's students, who now hail from a broad demographic and who are accustomed to acquiring their knowledge from a variety of different media, requires a new set of teaching tools. Using perspectives from four different immunology educators, we describe a range of student-centered, active learning approaches that have been field-tested in a number of different immunology classrooms and that are geared to a variety of learning styles. In this paper, we explore the hypothesis that active learning approaches to immunology improve comprehension and retention by increasing student engagement in class and their subsequent mastery of complex topics.

## Introduction

Not so long ago, immunology was regarded as a medical sub-specialty, taught exclusively to medical or graduate students and rarely offered at the undergraduate level. In contrast, today's undergraduate biology curricula frequently include one or more elective courses in immunology. Collectively, the co-authors of this article have taught immunology to undergraduates for more than 90 years. In the process, we have endeavored to keep pace as our field has matured to a discipline ripe with opportunities to integrate and contextualize many of the core concepts that students encounter in a standard biology curriculum. Simultaneously, pedagogical advances have encouraged us to expand the range of tools that we use to cultivate and assess student learning. This article will describe some of the ways in which we have employed active learning strategies to help students understand how the immune system works; a topic they report to be as challenging as it is fascinating.

Students enjoy studying immunology in part because it teaches them about their own bodies; indeed, it is often the first medically-relevant course they experience in college. In addition, they take delight in the subject as an integrative discipline that asks them to apply information learned in other courses (e.g., biochemistry, genetics, cell biology, anatomy, physiology, etc.), to the study of an organism-wide system. They also learn to appreciate that immunology is a dynamic field in which important conceptual advances are still emerging.

However, our students frequently struggle with the discipline's specialized and often arcane vocabulary. Just like learning a foreign language, a course in immunology requires students to learn the meaning of new words, and then rapidly apply that new vocabulary to build a knowledge base and answer complex questions. It is therefore not surprising that some students flounder or become discouraged in the early weeks. We have found that a flexible approach to the subject with use of creative learning strategies can help students overcome these initial hurdles.

In this paper, as four seasoned teachers, we share some of the approaches that have worked for each of us in the undergraduate immunology classroom. We acknowledge that immunology courses are content-heavy and as such, must include extensive reading assignments as well as some conventional lecture components. However, a considerable body of pedagogical research has shown that, if we want students to retain the material they are exposed to, apply it to future situations, and in particular, appreciate the connections between different sections of this course and between this course and others, we need them to engage in “*active learning*” (1).

What is “active learning”? A frequently cited paper by Bonwell and Eison (2) suggests that students participating in active learning “must do more than just listen: They must read, write, discuss or be engaged in solving problems. Most important, to be actively involved, students must engage in such higher-order thinking tasks as analysis, synthesis and evaluation.” They continue: “Within this context, it is proposed that strategies promoting active learning be defined as instructional activities involving students in *doing* things and *thinking about* what they are doing” (emphases ours). Thus, most active learning approaches are also student-centered, positioning the student as the architect of their own knowledge and building metacognitive skills. Active learning strategies can vary greatly, from short group discussions to more complex single- or multi-day engagements, and the effectiveness of particular approaches should be carefully and frequently assessed, with adjustments made as necessary (3). However, the use of active learning in the classroom has been clearly demonstrated to improve student understanding across all STEM disciplines (4). Importantly, the pedagogical literature further suggests that, although *all* students gain from the use of active learning strategies, those students from non-college preparatory backgrounds or who fail to thrive in standard lecture-based settings derive particular benefits. Thus, active learning approaches can be instrumental for leveling the playing field, and are therefore viewed as vital for effective and equitable teaching (5).

Current students have been born into an information-saturated environment; answers to even the most obscure question can be ascertained within seconds via any search engine. Therefore, instructors in the modern classroom must thoughtfully teach students how to distinguish between verifiable information supported by reliable evidence and random “search results” devoid of scientific support (6). Today's faculty are also increasingly challenged to work with a student population that tends toward impatience in accessing information and adheres to a “faster is better” philosophy (7). Many of the strategies discussed below attempt to address this issue, using critical analysis, reflective discussion and contextual placement of information, interleaving technology that feels natural to today's student.

Active learning involves moving the focus away from the instructor and toward student engagement, both inside and outside the classroom. The use of technology and electronic devices can provide important conduits to foster this engagement, especially in large classes. However, questions regarding the appropriate use of electronic devices in the classroom are bound to arise. Research has documented the potential for distraction when such devices are unregulated (8) and experiments have shown that students who take class notes in longhand show better retention than those who use a keyboard (9). Nonetheless, tablets and other electronic devices can be powerful tools to facilitate learning, especially given their ubiquitous presence in our lives. Approaches for the effective use of these devices in the classroom will be discussed.

In this article we describe a variety of interactive exercises that have been field-tested and found to work in our immunology courses. These include concept maps, “Just-in-Time” Teaching strategies, classroom games, formative writing assignments, reenactments of cellular events, written, video or audio material, as well as the use of tablets in the classroom. Throughout, we discuss the use of technology in ways that help draw students in rather than intimidate them, and we provide examples that can be used by interested instructors. We recognize that there are many potential active learning approaches that are not covered by this article. For example, immunology-based labs and the use of primary literature are not presented here, but instead are addressed by other articles in this volume (10–12). Likewise, the important discussion of how these approaches, and others, can help reduce learning inequities in the U.S. (13) and beyond (14) appears elsewhere in this issue.

We believe that our classroom experiences support the hypothesis that active learning approaches greatly assist students in understanding the complex and interdisciplinary discipline of immunology. We have found that, by teaching immunology with these techniques, our students demonstrate retention of material from module-to-module and critical thinking. We have taken a “before, during and after” approach to our description of the techniques. We begin by discussing some strategies that we have found help students to arrive in the classroom prepared to engage, such as Just-in-Time Teaching and video or audio preparation. We next address the involvement of students in classroom activities, including clicker questions, reenactments, strip sequencing, short presentations, and concept maps. With our discussion of the different kinds of concept maps and their varied use, we bridge into providing examples of work that students may begin in class but complete outside of the classroom, and then move into a description of some non-examination writing assignments. We finish with a discussion of novel uses of iPad devices in the teaching of immunology, both inside and outside the classroom.

## Pre-Class Preparation and Just-in-Time Teaching

Learning immunology requires engagement with new vocabulary and some paradigm-breaking biological concepts. We have found that immunology students who regularly spend time before class grappling with new words and concepts come to class more prepared to ask good questions, practice their skills, and apply this new knowledge in higher-level thinking endeavors. In our experience, if students participate in well-designed, pre-class preparation exercises, the in-class time with the instructor is demonstrably more productive. Furthermore, our students report that the additional workload imposed by these pre-class exercises is worthwhile, because it aids in their understanding through reinforcement of pre-class material in class, and helps to entrain an incremental work ethic as opposed to cramming.

We have found the method of Just-in-Time Teaching (JiTT) to be an excellent strategy for organizing and implementing pre-class immunology-based learning (15). Basically, JiTT is a form of homework reimagined or a semi-flipped classroom (before its time), employing regular pre-class exercises called “warm-ups” or “pre-class questions,” to motivate and direct student preparation shortly before class meetings (16). Students are asked to read, listen to a podcast, or watch an online video in preparation for answering questions shortly before class. They then submit answers to these pre-class questions online, between 1 and 24 h prior to the class meeting. Student responses are used by the instructor to provide whole-class feedback and to better focus the in-class plan on the collective needs of the students, transforming the classroom from instructor-dominated to student-centered (17). In this way, feedback in both directions occurs “just in time” to appropriately address the material at hand. Ideally, this creates a feedback loop where in-class and outside-of-class work is highly connected, where the instructor is consistently apprised of the level of student understanding and where students can identify faculty expectations for mastery. An added benefit for the instructor is that there is nothing better than walking into a class where students are already hotly engaged in a debate over their thoughts on questions related to that day's topic!

The structure of JiTT pre-class exercises or assignments can vary, from questions that probe basic vocabulary or the application of concepts, to real-world dilemmas or queries about an assigned journal article. Questions that highlight common confusions and misconceptions are ideal. We have found that a combination of recall or fact questions (especially early in the semester), along with some higher-level questions that require open-ended responses, provides a good mixture of positive reinforcement and challenge. Examples of pre-class questions related to two topics are shown in Table 1. Optimally, at least one open-ended question and an opportunity for students to ask the instructor their own questions are included in each assignment. Responses to open-ended questions, in particular, provide valuable, low-stakes opportunities for students to articulate their understanding of topics in their own words, using their newly-acquired vocabulary. Likewise, by adding “with rationale” to True-False or Multiple-Choice questions, students are given an opportunity to briefly explain their thinking.

**Table 1:** Examples of pre-class questions for just-in-time-teaching.

**Topic: primary vs. secondary responses and innate vs. adaptive immunity**
1. (True/False) Primary lymphoid organs are where lymphocytes develop and become activated. Please provide a brief rationale for your choice.
2. (True/False) Innate immunity involves soluble products and is a part of humoral immunity, while adaptive immunity involves the work of B and T cells, or cell-mediated immunity. Please provide a brief rationale for your choice.
3. (Essay) Is adaptive immunity engaged during both a primary and a secondary immune response? What about innate immunity? In other words, what is the relationship, if any, between the innate/adaptive and primary/secondary immune response?
4. (Optional) Do you have any questions from this part of the reading/viewing preparation for class? Please be as specific as possible.
**Topic: innate responses and pattern recognition receptors**
1. (Multiple Choice) Which of the following type/s of PRR/s are responsible for detecting foreign antigens in the cytosol of an infected cell? Please select all that apply.
A. TLRs
B. CLRs
C. NLRs
D. RLRs
E. ALRs
2. (Multiple Choice) Based on shared vs. unique properties, which two categories of pathogen do you think might be treated most *differently* by the immune response? Please provide a brief rationale for your choice.
A. viruses and intracellular bacteria
B. viruses and extracellular parasites
C. extracellular bacteria and extracellular parasites
D. fungi and extracellular parasites
3. (Essay) There are only a small number of different ligands, or different “types” of ligands, for TLRs (see Table X in your textbook). What patterns or common features do these ligands share? Thinking of evolution and natural selection, why do you think these types of ligands make “good choices” in terms of recognition structures for the immune system?
4. (Optional) Do you have any questions from this part of the reading/viewing preparation for class? Please be as specific as possible.

We suggest that most, if not all, of the credit for these pre-class exercises should be awarded for good-faith attempts to answer questions, or for a clear articulation, using immunologically accurate terminology, of any areas of confusion. The use of pre-class questions allows the instructor to come into class with a distinct sense of the parts of a topic that are causing students the most difficulty, as well as the overall level of student understanding. More importantly, the questions provide students with valuable opportunities to think through complex information in their own time. Some instructors award a fraction of available points for accuracy. In our experience this can be counter-productive, as it can lead to a focus on the one right answer over an explanation of reasoning that, even when flawed, may illuminate misconceptions and roadblocks to learning. At its most effective, JiTT affords instructors an opportunity to peek inside the heads of their students right before class.

JiTT can also be valuable as a term-long system for organizing the assignments and workflow for both faculty and students. This method has been shown to help spread the work of studying more evenly throughout the term and makes it much harder for students to fall behind without the instructor's knowledge (15). In 20 years of using this technique to teach immunology, students routinely report that weekly pre-class questions are one of their favorite parts of the structure of the course. Almost one half of students in a recent undergraduate course said that the questions ensure that they always know what they “need to know” and where to focus their attention while reading, and that this activity forces them to keep up with the material, minimizing the need to cram before exams.

This pedagogical strategy works best when the thinking students do before class is closely aligned with that day's material, and students are given immediate opportunities to either demonstrate mastery or identify their areas of uncertainty. Likewise, follow-up questions that arise during class can be included in the next set of pre-class assignments, setting up a nice learning feedback loop. While the design of good questions can be time-consuming at first, effective questions can become the material for the day, making planning for class time relatively easy. Finally, in our experience, the JiTT strategy is especially helpful for non-traditional or first-generation students and others who thrive in highly organized academic settings, where outside-of-class expectations are laid out clearly and where there are regular opportunities for low-stakes, formative assessment (13). It is worth noting that significantly more students from sections set aside for students from resource limited backgrounds (with no other course modifications) made favorable comments on the use of JITT than those in traditional course sections. We believe that techniques like this, where outside-of-class expectations are laid out clearly and there are regular opportunities for low-stakes, formative assessment, can help to level the playing field (13).

## Video and Audio Supplements

Technology enables the use of audio and visual tools for powerfully conveying information. Today's students are used to acquiring information from videos or by listening on their phones and other devices. While strategic reading and note-taking are invaluable exercises that allow students to integrate knowledge and develop their own interpretation of a topic, supplementing reading assignments with video or audio material is particularly useful when students are learning complex topics or reviewing background material. For example, transfer students may not have had a molecular and cellular biology course for several years. Reviewing the central concepts of cell and molecular biology will be essential for understanding certain immunological topics. We have found that high quality videos provide an excellent way for students to get up to speed before class.

Videos can also be used to introduce and illustrate new topics. “Seeing” complex pathways of cell interaction or protein cascades in action can help students better understand them. Table 2 lists some of the videos that we have found to be most useful. For example, several Khan Academy and Crash Course videos are particularly useful for both review and learning new material. Kurzgesagt–In a Nutshell provides entertaining cartoon animations that students enjoy. Videos from Nature, iBiology, HHMI, and the Walter and Eliza Hall Institute, are all high quality and highly illustrative, as are some of the short videos or animations offered as Supplementary Materials with various textbooks, research papers or on the websites of faculty active in particular fields or research^1^. Videos can be paired with worksheets or pre-class questions (see JiTT section) to ensure students focus on the concepts that the instructor will later reinforce in the classroom.

**Table 2:** A list of immunology video supplements.

**Video**	**Home: youtube or website**	**Sample video**
Crash course	https://www.youtube.com/channel/UCX6b17PVsYBQ0ip5gyeme-Q	https://www.youtube.com/watch?v=GIJK3dwCWCw&t=436s
HHMI biointeractive	https://www.biointeractive.org	https://www.biointeractive.org/classroom-resources/targeting-infected-cells-immune-defense
iBiology	https://www.ibiology.org	https://www.ibiology.org/online-biology-courses/immunology-flipped-course/
Khan academy	https://www.khanacademy.org/	https://www.khanacademy.org/science/biology/human-biology/immunology/v/role-of-phagocytes-in-innate-or-nonspecific-immunity
Kurzgesagt–in a nutshell	https://www.youtube.com/channel/UCsXVk37bltHxD1rDPwtNM8Q	https://www.youtube.com/watch?v=zQGOcOUBi6s&t=15s
Nature	https://www.youtube.com/user/NatureVideoChannel/featured	https://www.youtube.com/watch?v=5AXApBbj1ps&t=6s
Nature immunology	https://www.nature.com/ni/video	https://www.youtube.com/watch?time_continue=2&v=CXz6FVqPqHw
Walter and Eliza Hall Institute	https://www.wehi.edu.au/wehi-tv/animation	https://www.wehi.edu.au/wehi-tv/immune-system

Another useful resource is the expanding array of science-related podcasts. Podcasts range from general interest to specialized presentations of new findings and important papers. However, it is important to choose podcasts that are accessible to undergraduates, piquing their interest rather than overwhelming them. A list of the podcasts we currently find most useful can be found in Table 3. The American Society of Microbiology (ASM) podcasts describe many pathogens and their interactions with the immune system, and also include a dedicated “Immune” podcast devoted to current topics in immunology. The ASM podcasts are particularly useful in that they allow filtering for criteria such as the target audience (undergraduates are one option). As these weekly podcasts consist of discussions and critical analysis of the latest cutting-edge research, we have also found them to be an excellent commute-time resource to keep ourselves up-to-speed in selecting new research to cover in class. The American Association of Immunologists (AAI) also produces an immunology podcast, although these are directed more toward graduate and medical students, or professional immunologists. Podcasts often include interviews with the authors of the study being discussed, increasing student's direct access to scientists. This can be particularly beneficial when these scientists come from non-stereotypical backgrounds, expanding students' visions of who scientists are, as well as what they look and sound like. If a paper chosen for a class discussion is discussed in a podcast, the podcast can be a helpful supplement.

**Table 3:** Examples of immunology podcasts.

**Podcast and comments**	**Link**
Bite size bio (https://bitesizebio.com/) has a listing of some top science podcasts; not all immunology but all are interesting	https://bitesizebio.com/24598/our-12-favorite-science-podcasts/
American Society of Microbiology; several different series, can filter for level of audience, some in Spanish	http://www.microbe.tv/immune/
Audioimmunity; informal, low key, likely to appeal to undergraduates	https://player.fm/series/audiommunity
Journal of Immunology; too specialized for most undergraduates	https://player.fm/series/the-journal-of-immunology-immunocasts
Nature; a wide variety of science podcasts of general interest	https://www.nature.com/nature/articles?type=nature-podcast
“Talkin Immunology with BioLegend;” a high quality production from BioLegend	https://www.biolegend.com/podcast

Whether using videos or podcasts, it is important to remember that multimedia content is in constant flux. Links must be checked and searches conducted regularly, as new material is released frequently. In fact, students enjoy being enlisted in the effort to maintain up-to-date digital resources and indeed, one useful class activity is to have the students search for new videos or podcasts and report on which ones they think are most useful and why. As part of the exercise in finding new online material, they can be encouraged to update links to existing videos and podcasts.

We have also found that students enjoy using audio books on topics related to the class material. The book *Get Well Soon: History's Worst Plagues and the Heroes Who Fought Them* by Jennifer Wright (18) has proven to be a popular choice for student listening, and the chapters on smallpox, polio, and HIV are particularly relevant to an immunology course. The audio recording of the book is excellent, and the author's popular culture references make it particularly relevant and entertaining for undergraduates. Students can either listen to or read assigned chapters (they are required to listen to the first chapter) and then write a short reflection paper. In one case, students were asked whether they preferred reading or listening to the material, forcing them to think about how they best acquire and absorb information and providing them, and the instructor, with important metacognitive feedback. Not surprisingly, the students had a variety of preferences: reading only, listening only, or reading while listening.

## Clicker Questions

Interactive response systems, more commonly referred to as “clickers,” have been used in educational settings for over a decade, as a way to engage students and encourage active class participation (19). During lecture, a question, most commonly multiple-choice, is posted by the instructor and students respond using a dedicated device (typically termed clickers) or via Wi-Fi on their own phone, tablet, or computer. Many students prefer to use their own Wi-Fi devices, which can save time and money (20). However, even when required to purchase an eClicker, our students consistently rank Clicker questions and follow up discussions as a favorite element of the course in helping them to assess and focus their learning.

While most clicker questions are framed as multiple-choice questions, the options can be expanded. Poll Everywhere, a web-based response system, includes word clouds, Q & A, clickable images, surveys, open-ended and even competition questions. Even when Clicker questions simply probe lower-order Bloom's skills like immunology vocabulary retention, the use of these questions at the beginning of each lecture provides students with the motivation to review the previous class before the subsequent one, which our students also report to bolster their incremental study habits. They also work well during lecture, first to review the terms or complicated concepts just introduced, then as higher-level questions that challenge students to build upon what they just learned. Textbook test banks, as well as instructor's homework and exams from previous courses, provide a good source when starting to use clicker questions.

The anonymity of clickers enables participation from students who might otherwise hesitate to verbally answer questions in class. Thus, clicker questions can provide a powerful tool for engaging students who are typically less confident about raising their voices in class, a group that frequently includes students of color, women, and those who may feel hesitant about their knowledge of the material (21, 22). By awarding points for *participation* in answering clicker questions, rather than for *arriving at the correct answer*, students are encouraged to answer questions when they are unsure and to take risks with their responses, providing valuable low-stakes assessment. Using a musical theme (such as the Jeopardy theme) as a timer adds an element of fun.

If the distribution of answers shows that many students are confused, we then utilize a Think-Pair-Share approach (23). In the case of clicker questions, the “Think” step corresponds to the students' original response to the question. Students then “Pair” with their neighbor, discuss the topic for a minute or two, and then “Share” by answering the question again or by participating in a whole class discussion. This approach is generally well-received by students and usually results in a notable improvement in comprehension. The discussion time can either be pre-set or left to the judgment of instructor. Students quickly learn that when the projected answers show significant variability, it's time to turn to their neighbor and discuss it. As instructors, we have learned that in the rare cases where we don't observe improvement after the “Think-Pair-Share,” we need to re-approach how we explain the topic. Clickers therefore serve as an excellent formative assessment for faculty to reflect on their teaching effectiveness.

A related fun activity that is also useful as a study tool is to create a Jeopardy game. One site, Factile^2^, allows instructors or class members to easily build Jeopardy style games. In addition, there are a number of games created by other instructors, which can be accessed by searching for “immunology” on the Factile website. Downloaded questions can be used by groups in class, employed individually as flash cards or as a memory game, or used by peer mentors during study sessions.

## Simulations, Reenactments, and Other Interactive in-Class Activities

Nothing makes a learning environment more active than when students get up out of their seats and move around. We have found that some elements of immunology learning are particularly amenable to simulations, reenactments or other interactive in-class activities. Students who struggle with auditory, visual or written modes of learning particularly benefit from activities that include kinesthetic elements. Most of these approaches also work well in study groups outside of class or during peer mentor sessions, and students frequently request access to the props outside of class. Below we briefly describe four examples of such activities. Most will require at least 30–45 min to complete, although timing can be varied by supplying more or less directed guidance and by adding discussion afterwards.

### Table-Top or Whiteboard Simulation of Somatic Recombination

V(D)J recombination is frequently cited as one of the most difficult concepts for immunology students to grasp; the structure-function elements involved in antigen-specific receptor generation and maturation as well as the use of multiple gene segments to create a single receptor chain are common sources of confusion for students. Paper or other props, including yarn “DNA,” can be used to depict the genomic arrangement of gene segments and their behavior during somatic recombination.

Groups receive packets containing V, D, and J gene segments, with multiple gene segments of each type. First they organize the segments as they would appear in a non-immune somatic cell. Next, students begin the process of recombination. Different colors are used for V, D, and J segments, and students are given multiple, numbered segments of each type/color, allowing groups to generate different receptors. The goal is to produce an arrangement of gene segments that encodes a TCR or BCR locus. Finally, students attempt to draw out their BCR or TCR as a protein on the cell surface, using colored markers linked to the colors of the paper gene segments to depict locations of the V, D, J, and C gene segments. This is a nice reminder of important gene structure-function relationships and can lead to discussions of somatic hypermutation and affinity maturation in B cells. If there is time remaining, students can walk around the room to see what other groups have produced, an activity that often leads to interesting discussions.

### MHC Diversity Illustration Using Simulated Genotypes and Phenotypes

When students and faculty are asked which aspects of immunology they find to be the most challenging, the MHC often comes out on top. For this reason, we continue to explore creative ways to present this particular topic. The following activity aims to help students grasp the difference between polygeny and polymorphism, as well as highlight how codominant expression allows unique class II allotypes to be expressed. Finally, this exercise illustrates the power of diversity at the MHC, and how this is manifested at the population-as compared to the individual-level.

**Figure 1:**
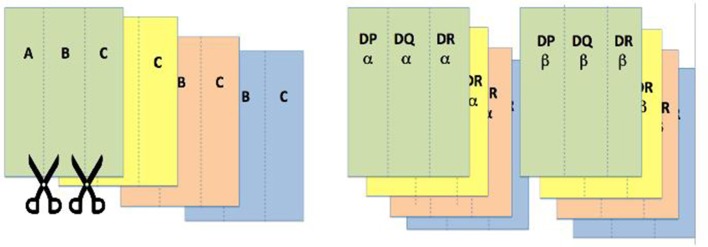
MHC Diversity Activity. Examples of the paper cut outs used to generate a “gene bank” for the MHC diversity activity. We usually make many copies of the same gene in the same color to represent common alleles, and one or a few unique colors for each gene to demonstrate rare alleles.

For this activity, colored paper props are used to depict alleles of MHC genes. Gene names are written on each strip of paper (e.g., “Aα” if using human, or “Kα” if using mouse, Class I nomenclature). The same is done for the class II region, with alpha and beta chains represented by separate strips of colored paper. See Figure 1 for a visual illustration of the props. The final product represents the theoretical diversity of MHC alleles in a given population. In class, students work in groups to create an individual genotype. This can be done either by providing each group with a packet containing all the genes needed for one individual (so, two copies of each gene) or by asking a representative from each group to collect from a “gene bank” what they need to depict the MHC genotype of one individual. At some point, students need to grapple with the presence of two copies of each gene due to maternal and paternal contributions and the presence of multiple genes of the same class. Non-classical, non-polymorphic MHC genes such as CD1 and MR1 can be included, with only one option for the strip colors, to provide a complete overview of the MHC locus.

Once their colorful paper genotypes have been laid out, students use them to create MHC molecules, and call an instructor over to check their work. Next, each group is asked to assume these slips of paper are protein chains and hold up in the air all the Class I molecules that would be expressed on the surface of this individual's cells. This usually leads to some questions regarding codominant expression. By looking around the room, we get a quick glimpse of individual diversity (e.g., some “individuals” have six colors of Class I molecules and others have only three) and population level diversity (a rainbow of Class I molecules in the room). This gets even more complicated when they are asked to do the same for all the Class II molecules expressed by their “individual,” as they deal with combinatorial association and questions of which chains can and cannot pair. Students can also be asked to hold up all those molecules expressed by different cell types, a question that asks them to recall the difference in expression between Class I and Class II MHC genes.

Groups are next asked to create the MHC genotype of a gamete for their individual. Groups hold up a single set of MHC genes for their gamete and then select another group to “mate” with. Pairs of groups now create the diploid MHC genotype of their “new individual” or progeny, at which point each group is asked to display the virtual MHC genotype/phenotype of their new individuals. Questions about whether new Class II molecules can arise from unique combinations of the alpha and beta chains originating from different parents provide important and productive opportunities for correcting misconceptions. Again, a glance around the room provides students with a sense of individual and population level diversity. An optional final segment of this activity involves posing questions about what happens when there are allelic associations with disease susceptibility or resistance connected to specific alleles (colors, in this case). This physical demonstration of the difficult concepts of polygeny and polymorphism in the MHC is consistently highly rated by students.

### Strip Sequence Activity to Practice Cell-Cell Interactions During an Immune Response

The many steps and players in the various cell-cell engagements that occur during a given immune response are notoriously difficult for students to master. Strip sequences or other types of poster materials are an excellent teaching tool in these instances. For example, the instructor can create a set of paper strips, each outlining an individual event, surface marker, intracellular component or signaling event; instructors can tailor the number of these “immune players” to the complexity of what they expect students to learn. Each student team is given a complete set of these paper strips, each of which also contains a number for tracking purposes (out of order, of course). The instructor provides one challenge question to the whole class, such as the following; “Map out the events that occur after a naïve T cell encounters cognate viral antigen, ending with an activated CTL.” The students use the paper strips containing immune players and events to create an appropriate sequence.

In one recent variation on this technique, students were asked to make a poster that described all the steps in an immune response from recognition of an antigen by a dendritic cell to the production of antibodies. Strips and cutouts included paper shapes representing cells, molecules and organs, not all of which were relevant, so students had to decide which cutouts to use and which to discard. Each group of four or five students received a large sheet of paper, tape, scissors, construction paper, markers and strips. The instructor circulated among the groups and answered questions. Students enjoyed the “big picture” aspect of this class and interesting questions asked by one group were shared with their colleagues. Some of the posters were visually stunning, but most importantly, this exercise allowed students to integrate a great deal of material in a very short period of time. In one recent class, 36% of the students highlighted the poster exercise as particularly useful in helping to synthesize the content of multiple lectures. Thirteen percent of the students were not enamored of this project.

We find that discussions of differing opinions can be very fruitful. For this reason, allowing time for a whole class discussion is quite valuable. By creating a really comprehensive set of strips and cutouts, instructors can reuse the same set and pose different challenge questions. This is also a great activity for outside-of-class review and mentor sessions, where the instructor supplies a list of challenge questions and a matching numerical sequence key for each.

### Reenactment Activities of T Cell Activation and B Cell Affinity Maturation

Reenactments of immune events using student volunteers and props are always a crowd-pleaser. Students who do not volunteer to come to the front of the room can still be engaged to direct the actions of others, so that everyone is involved. For large classes or to encourage discussion by more reluctant students, this direction can be provided using student audience teams. One example of this activity is a reenactment of T cell clonal selection in a secondary lymphoid organ. The instructor prepares by bringing required props and labels. We like to use candy (e.g., Hershey's Kisses) for the antigen, chairs, and tables to depict particular microenvironments (e.g., follicle or a follicular dendritic cell), and student volunteers to act as specific cellular players. These volunteers wear signs around their neck declaring their cell types (e.g., dendritic cell, B cell, T Helper cell, etc.). The instructor can also provide multiple clip-on or pin-on buttons for relevant surface markers (e.g., CD28, B7, CD40, etc.), asking the students to determine when and where these are needed. MHC molecules can be labeled plastic cups, which hold peptide fragments. Decoy surface markers make a nice challenge and help identify important misconceptions.

Students in the audience are asked to direct the action by providing the student “cell-type volunteers” with instructions regarding what to do and where they need to go at each step. In this example, the action might begin as the dendritic cell encounters antigen in the periphery or in a secondary lymphoid organ. Students in the audience tell the cellular players which buttons they will need (e.g., maybe a particular pattern recognition receptor), when they need to acquire these and how interactions with other cells should proceed. For example, the “dendritic cell” student might use a pattern-recognition receptor button to acquire antigen (e.g., Hershey's Kisses). The antigen must then be processed (make sure this volunteer likes chocolate!) and “presented” as a fragment (the Hershey Kiss label works well for this), along with an MHC class II molecule (labeled plastic cup holding the Hershey Kiss label). This MHC-antigen fragment is presented to the student acting as the T cell, along with a B7 button in the other. The T cell student volunteer is told to respond using one hand as the TCR and holding the CD28 button in the other hand. And so on.

For the affinity maturation enactment, T-B cell cooperation and sequential rounds of somatic hypermutation can be simulated using additional student “cell” volunteers. The process by which T and B cells recognizing the same antigen can join forces, even when recognizing different epitopes, soon becomes apparent–a topic that is notoriously challenging for students to grasp. Affinity maturation can be simulated using additional students acting as B cell progeny, with higher or lower affinity receptors, following rounds of somatic hypermutation. To depict the evolving process of changes in BCR affinity, the “progeny” selects from a deck of cards labeled “higher affinity,” “lower affinity” or “same affinity.” The higher affinity B cell player gets to use all five fingers and both hands to pick up as much candy as possible from the table (a follicular dendritic cell). The player with the “same affinity” card is told to use only thumb and one finger of each hand, while the “lower affinity” B cell player is further handicapped in some way from grasping candy, simulating affinity maturation. We then count the candy acquired by each B cell player and discuss what happens next in interactions with T helper cells or as antigen becomes limiting. The final discussions of this activity can be done as a whole class, by engaging table groups or as an after-class assignment if time runs short. This activity can take most of a class period but is one of the more effective ways we have found for driving home this process, complicated by multiple cell types, surface molecules, events and locales.

## In-Class Short Presentations and Posters

In information-dense courses like the average undergraduate immunology class, students rapidly come to appreciate a respite from listening to their instructor and gazing at PowerPoint slides, no matter how accomplished the professor or how appealing the images. One technique that has proven useful has been to divide the course or the lecture day into sections, with the faculty member sharing some in-class presenting time with students. For example, the instructor might set the stage for a topic and then have groups of students deliver some of the material. Sometimes, the faculty member will need to return to emphasize the main points that have been made and offer a summary of the conclusions. Alternatively, students can be charged with presenting on a topic of interest to them, as it relates to immunology.

This approach is particularly useful when the subject involves presenting information in the form of a list of similar, but slightly different molecules, cells or even topics. When student groups are responsible for different parts of the lecture the perceived “sameness” of the material is broken up, helping the students to associate particular molecules with different people, and acting as an *aide memoire*.

In one example, the instructor introduced the overall concept of innate immune receptors and then delved into a discussion of members of the TLR family. Student groups then took up the story of innate immune receptors, with groups of three or four students presenting the other innate receptor families, while a separate group tackled the inflammasome. The structure and function of different cytokine receptor families is another topic that works well in this approach. Designing and delivering these mini-lectures gives the students (usually much needed) additional experience and confidence in presenting to a group.

Another popular student-led classroom activity is a variation on the classic paper presentation. A more detailed discussion of how research articles and reviews can enhance the immunology classroom is to be found elsewhere in this volume (12). All the students in the class are given a paper to read, but a subgroup of those students is asked to prepare a set of questions about the paper for their peers. The questions are shared with the instructor ahead of time, who works with the subgroup to refine the questions and then to select two or three of the most interesting for class discussion. This activity typically takes 20–30 min of the class period. Moving away from a rote presentation that lists all the figures and tables in turn, but instead asking specific questions that students must address, ensures a deeper level of engagement on everyone's part and better models the scientific process.

In some courses, a segment of the semester, usually near the end, is dedicated to short student presentations. Since many of our students enroll in immunology with specific interests or connections to immunologic disorders, allergies or immune therapies, saving time for students to engage in self-directed learning can be rewarding for all. This gives the students a chance to practice their vocabulary and concept comprehension, plus some ownership over their learning. Since the instructor is released from class preparation during this time, they can meet with individuals or small groups to discuss content, structure and presentation style, improving the experience for all. Likewise, a requirement that students include some data from the primary literature in their presentations gives them a chance to practice reading about, interpreting and presenting scientific results. To lower stress levels and increase overall performance, clear guidelines and a grading rubric published well in advance are vital. In fact, these rubrics can be adapted for fast, real-time grading and feedback, making assessment less onerous.

Finally, in-class student presentations can be converted into poster sessions, especially helpful when class sizes are large. Posters can be generated by small groups or individual students, and can cover specified topics or areas of student interest. Again, clear guidelines and grading rubrics are important. Students can hang their posters and the class can enjoy a “gallery walk” around the classroom to learn about what other students investigated, with or without formal evaluation. Grading can also be done by scheduling meeting times with individuals or small groups of students, where they “present” their poster for Q&A and evaluation outside the usual class meeting time with the instructor. Downtime during associated laboratory sessions can work well for this purpose.

## Concept Maps

When studying any discipline for the first time students lack cognitive “hooks” on which to hang the new facts and ideas they encounter. Lacking a framework within which to organize their new knowledge, students can become overwhelmed and feel like they are swimming in a sea of unrelated factoids. Students of cognitive psychology will be familiar with the work of David Ausubel who showed how important a student's prior knowledge is to the acquisition and processing of new, related information (24). Building on Ausubel's theories, Novak and his research team developed the methodology of concept mapping as a means by which science students at all levels could position newly-acquired information in a pre-existing knowledge structure (25). We have found that our students are very polarized in their assessment of concept maps on course evaluations; however, enough students report finding them extremely helpful in synthesizing and modeling their recently gained knowledge to merit their continued use in our classes.

**Figure 2:**
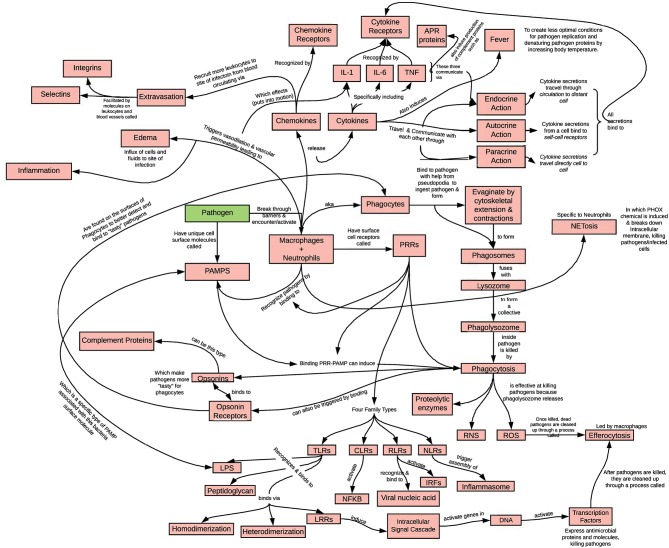
Example of a group generated concept map. An example of a student concept map of innate immunity and inflammation, using the terms listed in Table 5. At the end of each week of class, the instructor assigns a list of important terms from that week for students to concept map, working in groups. One such concept map developed by four students working outside of class is shown. Each boxed term was on the list of assigned terms. The instructor grades the map for accuracy and completion. Students generated this concept map using the Lucidchart program, which has free educational access and allows real-time collaboration between students.

A concept map typically represents each idea, experimental result, organ, cell or molecule as a shape, joined to other shapes by lines that indicate the conceptual connection between them. These lines can be labeled with phrases that are used to describe the relationship between the linked shapes. In a classical concept map, such relationships might be, for example, “causes,” “requires,” “combined with,” “is part of,” “occurs simultaneously with” or “occurs within” (see Figure 2).

We have found that this technique is particularly well-suited for teaching immunology, as students must master an impressive number of new words and concepts in a short period of time in most introductory courses in the discipline. However, like learning a foreign language through immersion, words and concepts are learned better when placed into their natural context and not simply memorized from index cards. We have had considerable success assigning concept maps after studying chapters that are particularly jargon-heavy. Concept maps work well as in-class group and individual activities, as well as take home study tools. Assigning students to work on creating concept maps in groups of three or four decreases the grading burden, allowing the instructor to give more thoughtful feedback to students; it also allows students the benefits of discussing with each other what belongs where, and why. Thus, in the process of making the map, the students are highly engaged in peer-instruction and metacognition.

Concept maps are most effective when there are multiple descriptor words on each arrow, and when many connections are made from a single node (26). Accordingly, we often find that the “messier” the concept maps appear, the better! Furthermore, the maps provide the instructor with a great assessment tool to identify where misconceptions lie or where connections were not made (27).

Concept maps make an excellent in-class activity, and have the advantage that they can easily be photographed and uploaded for assessment. These maps, whether handwritten or electronically-generated, can be saved as photograph or image files and later uploaded for grading. Especially when generated by hand, these maps can be quite creative, allowing students to express their artistic skills. Figure 2 shows a concept map generated with the Lucidchart program, which has free educational access and allows real-time collaboration between students. Table 4 lists the terms used for the complex concept map shown in Figure 2, while Table 5 lists terms useful for an in-class exercise. Individual concept-mapping exercises require students to organize the information on their own, which can be helpful for students who find speaking up in a group to be challenging. Making or studying previously-made concept maps can be an excellent review tool for certain types of learners.

**Table 4:** Terms for the concept map shown in Figure 2.

**Terms to be used in your innate immunity and inflammation concept map**	
Pathogen	Inflammation
Integrins	Phagocytes
Selectins	Cytoskeletal extension and contraction
Extravasation	NETosis
Chemokine receptors	PRRs
Cytokine receptors	Macrophage
APR proteins	Neutrophil
IL-1, IL-6, and TNF	PAMPs
Fever	Complement
Endocrine	Opsonin
Paracrine	Opsonin receptor
Autocrine	Proteolytic enzyme
Cytokine	RNS
Chemokine	ROS
Edema	TLR
Heterodimer	CLR
LRRs	RLR
NFkB	NLR
Viral nucleic acid	LPS
Intracellular signaling cascade	Peptidoglycan
DNA	Homodimer
Transcription factor	IRFs
Efferocytosis	

**Table 5:** Sample concept map terms for an in class exercise.

**Terms to be used in your BCR-TCR concept map**	
Lymphocyte antigen receptors	Ig domain
B cells	Igα
T cells	Igβ
APCs	CD79α
MHC Class I	CD79β
MHC Class II	TCRα
BCR	TCRβ
TCR	CD21
Antigen	CD19
Antigen peptides	CD81
Variable regions	CD3γ
Constant regions	CDδ
Heavy chains	CDε
Light chains	CDζ
λ light chain	ITAMs
κ light chain	CD28
CDRs	CD80 or 86

Assigning concept maps for group work outside of class brings students together to discuss the material and facilitates the formation of study groups. However, finding time to spend on group work each week can be particularly challenging on commuter campuses. In this case, students have utilized creative strategies such as skyping with cameras aimed at whiteboards, or completing the maps individually followed by a group meeting to share and critique each other's maps, and then develop one to submit as a group. An advantage of this layered process is that, as students notice aspects of the maps generated by their colleagues that are different from their own maps and discuss how to insert the new content into the master map they are actively synthesizing ideas and making new connections.

**Figure 3:**
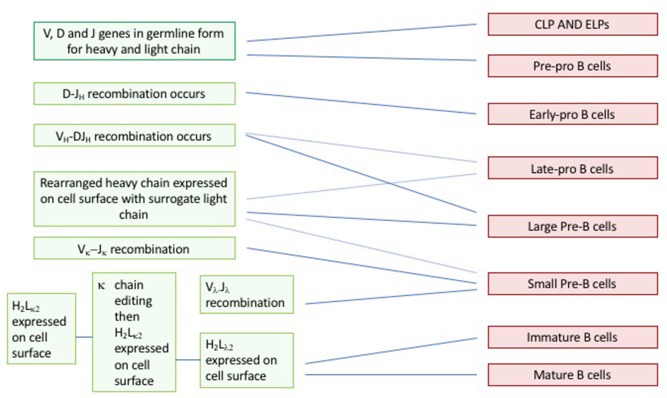
Student-generated B cell development concept map. B cell development concept map. Working in groups of two to four, students arrange the sticky notes on a poster chart in two vertical lines, representing the chronological sequences of events or differentiation states. Next, they draw the horizontal lines that connect the stage of gene rearrangement with the stage of B cell development.

Another use for concept maps is to help students better visualize and connect the sequencing of steps in pathways that occur on different conceptual levels. For example, students usually learn about the genetics of V(D)J recombination and B cell development in different lectures and with reference to different textbook chapters. A concept map done as a physical exercise after students have learned about the two processes can provide a useful tool to line up the two sets of ideas to create a picture of what is happening when and where, as shown in Figure 3.

In this example, students are provided with, or prepare themselves, two sets of paper strips, each set in a different color. One set contains the steps in V(D)J recombination, e.g., D-J_H_ joining; activation of TdT; V_L_-J_L_ joining; cell-surface expression of pre-B cell receptor; etc. The other set contains descriptions of stages of B cell development, e.g., pro-B cell stage, small pre-B cell stage etc. Students arrange the colored notes in two vertical columns, with one column showing the sequence of gene rearrangements and the other, the sequential development of B cell precursors. Students then draw lines between the two columns to show the correct relationships. This exercise is easier if the paper strips are sticky notes, as they can be arranged on a white board or poster, so that students can easily compare their maps. For a further challenge, an additional set of paper strips can be included that lists surface molecules such as CD79, CD19, pre-B cell receptor, etc. (not shown in Figure 3).

This mapping exercise allows students to develop for themselves a picture of what is happening at each stage of B cell development. If half of the class works on T cell development and half on B cell development, a comparative discussion can follow. Yet another variation would be to ask students to add a third column describing the place in the body and/or within the immune organs where the relevant cells are found.

## Writing Projects in Immunology

John Bean's masterful book, “Engaging Ideas” (28), stresses the importance of carefully-designed writing assignments to the development of students' critical thinking skills. In her foreword to this book, Maryellen Weimer emphasizes that: “Writing forces the clarification of ideas, attention to details, and the logical assembly of reasons.” In this section, we offer some examples of writing assignments that we have used successfully in our classrooms. Since each assignment requires students to perform some level of independent library research, students may benefit from interacting with library staff early in the course of each project, possibly during a class or lab period, so that they can master a range of specialized scientific search methodologies.

Of course, building up the skill of scientific critique takes some practice, especially when students are new to the primary literature. Attention to this before assigning writing projects can therefore be beneficial. One appealing example of a short writing exercise that aims to build critical thinking and break students of their reliance on the conclusions made by others, involves providing them with a “naked paper”–fragments of a research article, usually just the tables, figures and legends, plus any details or abbreviations they need to understand the data as presented. The students are then asked to write a short abstract and a proposed title for these data (on their honor not to try to just look this up!) outside of class. The instructor then shares the real paper, for comparison, along with anonymous student abstracts, maybe with a vote for the winner who came closest. After that, it's valuable to spend some in-class time discussing what story the students believe the data actually told, how the authors choose to interpret their data, and whether sufficient results were presented for the conclusions the authors made. Of course, it's important to select the paper for this assignment carefully. We've found that short research articles requiring only moderate background, especially those that lend themselves to alternative conclusions, can help students to build this critiquing skill and lead to excellent discussions.

### Treasure Trail / Biography of an Experiment

In the simplest form of this assignment, the Treasure Trail, students working in small groups select a seminal paper in the field and begin by learning about the original rationale for the experiment. Why was the question important? What was already known at the time the paper was written? What was the hypothesis/hypotheses being tested and what was the major advance described in this paper? Students are asked to explore the methodology of the experiment in depth so that they fully understand how the data were generated. They look up anything they don't understand about the experimental techniques, data presentation and analysis, and critically evaluate the conclusions.

Members of the group then pool their knowledge to develop a series of questions designed to lead a beginning student through the important points of the paper, and write the answer key. Asking the students to write a guide to the research paper in a question-and-answer format requires them to achieve a high level of clarity in their understanding of the paper, and working in a group context helps to ensure that students are not frustrated in isolation by the complexities they encounter. Details about this assignment are provided at the beginning of the course and they are given a considerable amount of time in which to complete it, in order to minimize time pressure and maximize opportunities for critical thinking, group work and for submission of revisions for grading. All students in a group receive the same grade.

The Biography of an Experiment assignment is a more complex variation of the Treasure Trail, and is particularly effective when used with senior students in small classes. (Development of “The Biography of the Experiment” concept owes much to the pioneering work of Profs. Jenni Punt and Iruka Okeke, both formerly of Haverford College). Students again are assigned a paper and perform the same type of research regarding its rationale, the experimental protocols that were used, the methods of analysis and data presentation, and the conclusion. The difference between the two types of assignment is that, in the Biography of the Experiment, students annotate the pdf of the paper to provide explanations of rationale, methodology, results, implications, critique and information about the authors as clickable links on the article itself. Where possible, students are encouraged to interview the senior author, either in person, or via phone or video link, and the annotated paper and interview are published together on the course website, along with a commentary by the student. The opportunity to communicate directly with a scientist who made a major contribution to the field has proven to be a particularly inspiring aspect of this project. This project can also be performed as a stand-alone independent study for an upper-level student.

### Boxes

Most biology textbooks now include “Boxes,” or discrete sections of text that are narratively separate from the main part of a particular chapter, but address related subject matter. In the textbook co-written by two of the authors of this article (SS and JO), boxes are classified into four categories: the evolution of some aspect of the immune system, significant advance, related clinical issue, or classical experiment. The key to this assignment is that a good box provides succinct, interesting and useful information about an immunologically-relevant concept that is of particular interest to the student. A typical box assignment will have an explanatory title, might be 800–1,000 words long, and includes at least one figure which may be derived from an original article or review paper, plus figure legends and attribution [see **Kuby, 8th edition** for examples of the different types of Boxes (29)]. Figures may be used as published or modified by the student to better suit their purposes (again, with appropriate attribution). Figure legends must be written by the student and show how the figure is related to the text.

Students typically enjoy Box assignments because they appreciate the opportunity to select a topic they are interested in and explore it in depth, developing their research and writing skills in the process. In our experience, many students use the chance to study a clinical application of an immunological topic, but of interest is that, as climate change becomes a more important part of a biologist's curriculum, plant immunology recurs more frequently among student topic choices, something that we encountered only rarely in years past. Faculty should take every opportunity in class to point out topics that they think might make suitable boxes, so that students develop a good sense of the range of options compatible with this assignment.

### The Mechanism of Action of a Drug That Acts on the Immune System

Many of our students plan to pursue a career in the medical or public health fields. Time constraints during a typical undergraduate course often preclude delving deeply into clinical aspects of the subject, and a written assignment that asks the student to explore a medically-relevant topic is often received with enthusiasm. There are many drugs that affect the immune system, as well as a range of pharmaceuticals derived from monoclonal antibodies, cytokines etc., that are designed to treat various malignancies and other disorders, such as hepatitis. In this assignment, students work in groups to ascertain the biochemical nature of the drug, its target and mechanism of action, and the disease that it is designed to treat. The results of the group's research can be expressed either in a short paper with appropriate figures and citations, or as a poster or PowerPoint presentation to be shared with the class. Almost all students highly rate this project, citing their enjoyment of the freedom to explore immunological mechanisms in a clinical context and the stimulation of working together as a group on a single paper. Those who did not enjoy the project often cited the difficulty of writing as a group and creating a cogent whole.

### Research Paper

Another approach is a research paper assignment that builds on the skills students acquire while learning to read complicated journal articles. Students identify three recent (published in the past 3 years) journal articles from different labs on a topic of their choice. These topics are often clinically related and need not all share the same conclusions. In their paper they introduce the topic, then analyze and critique each paper in turn. Next, they synthesize the findings of these papers, build a model explaining the results, and propose future experiments. Alternatively, students can elect to investigate research papers from 2 to 3 different decades in our evolution of understanding on a particular subject (e.g., tolerance) or a sequence of papers from the same lab describing a progression of ideas on a specific topic (e.g., regulatory T cells).

Students write this paper in stages, first getting their topic and papers approved. They then submit their first draft for peer editing by two other students. The peer editing consists of both written feedback and in-class discussion. For the in-class discussion, students meet in groups of three, with two students discussing the third student's paper for about 15 min. The written comments can be shared as a hard copy or uploaded to a class Google Drive. Peer reviewers are expected to go beyond editing comments, and critique the scientific content and clarity of the paper. This face-to-face peer review is very effective, and provides another opportunity for improving communication skills.

The paper can be a major assignment in the class, with the majority of the points given for the final product, although students receive some points for their first draft (to ensure that it is complete) and for the quality of their peer editing. Originally the first drafts were also edited by the instructor but students tended to address the instructor's points and ignore the peer review. However, students are encouraged to meet with the instructor to discuss their articles, and almost all do so.

### Critical Analysis of Social Media Posts

The hot topic of immunology appears often on social media posts, where much of the population gets its news. For many instructors, fostering the ability of students to critically evaluate immunology as it appears in the world around them is a major learning objective. As students progress in their learning and acquire the skills to read immunology articles, they are well-positioned to read social media reports and immunology news stories with this critical eye. For this activity, students are directed to pay attention to science-related social media feeds throughout the term, choose a post related to immunology that they find interesting, and then research the accuracy of the post, using primary literature. The student then writes a paper summarizing what the primary literature shows about the topic, comparing it to what is reported or conveyed in the social media post, and then evaluating whether the social media post was accurate and responsible. This exercise gets at higher-order Bloom's taxonomy skills (evaluation and critical thinking) while utilizing a space in which many students spend much of their time, on social media, further reinforcing the real-life relevance of course content. It also encourages students to become peer-educators, commenting on each other's posts.

## iPADS as a Tool For Active Learning

While electronic devices are often seen as an unwanted usurper of student attention in the classroom, we believe this equipment can be used to promote active learning. In particular, iPads are being increasingly used in classes at many levels (30–32) although they are not a ubiquitous feature in the classroom. The most often reported uses in undergraduate science classes are for Anatomy and Physiology, which tend to be particularly image-intensive (33, 34). Given that today's students are digital natives and use electronic devices as a regular part of their daily lives, tablets such as the iPad are comfortable and intuitive for them to use.

One of us (RP) has successfully incorporated iPads into teaching immunology (31). Each student in the course is issued an iPad for the semester, which is used in multiple ways throughout the class. Providing iPads to the entire class ensures that all students, regardless of financial need, have access to this technology. Students are expected to determine which of the available iPad tools and apps work best for them, requiring reflection on their own learning styles, and to adopt the approaches that best help them learn the material, building their metacognitive intuition.

What advantages do iPads offer over a laptop or paper and pen? The devices are portable and lightweight, the touch screen allows easy manual manipulation, and there are many apps that offer fun new features not available with more conventional tools. As students increasingly prefer to submit work electronically, the ability to complete and immediately upload forms, worksheets, etc. on the iPad, either written by hand or typed, is also highly attractive.

Likewise, using iPads in class allows students to project their work to the class or as part of a class presentation, and to submit this for assessment. The camera feature is also useful, to copy complicated illustrations that are drawn on the board by the professor or other students, and to document hands-on work done in class. As students increasingly rely on videos as learning tools, iPads also provide a convenient vehicle for this. In our hands, distraction during class has not proven to be a problem with this approach, especially since students generally would have laptops or smartphones in class anyway. Plus, an iPad allows students to organize the material for their class, and takes much less space in a backpack than accumulated handouts, a notebook, laptop, and textbook.

iPads can also provide specialized help for many students with disabilities, who use design features built in for individuals with vision or hearing disabilities (35). One student with a movement disorder found the iPad to be particularly helpful, and subsequently purchased one to help her in her graduate studies in neurobiology. Lectures can be recorded and linked to a PowerPoint (see the Notability app below), which is useful for students who normally need note-takers or record lectures with other devices. The ability to enlarge what is on the screen, whether text or images, as well as record lectures and synchronize the recording to notes, are useful for all students, but especially for those with learning differences.

Certain iPad apps are particularly useful in academic settings and the ones we use most are listed in Table 6. One such app is Notability^3^. The most common use is for notetaking; lectures (PowerPoint or other formats), journal articles, or other materials can be uploaded as pdfs, and notes taken directly on the pdfs. A fiber mesh stylus or Apple pencil allows for the most fluid writing. Notability is particularly useful for lectures, as the record function links with the handwritten notes, allowing students to go back and review what was being said with a particular slide. In our experience, virtually all students utilize the record function. (The responsible use of the record function does need to be covered when the iPads and Notability are introduced to the students). There are numerous color options and line widths for writing, highlighting and typing, allowing students to organize their work using color and emphasis. The automatic saving function and easy sharing of notes are another big plus. Students can also use the Notability app to take photos during lecture of drawings on the board, which are then seamlessly incorporated into their lecture notes. This app is the most extensively used one in the course, and the one that students are most likely to use in other classes.

**Table 6:** iPad apps useful in immunology classes.

**App**	**Purpose**
Notability	Note-taking, writing, drawing
Poll everywhere	Clicker questions, with multiple types of questions
Google drive	Shared file access
Dropbox	Shared file access
RCSB protein data bank	Visualizing proteins of the immune system
BioLegend	Provides useful information and tools
Inspiration	Concept mapping

Discussions of original journal articles are an essential component of most immunology classes, and Notability can be valuable tool here as well. Students often comment that Notability allowed them to easily mark passages or add comments in articles or other students' papers for later discussion, and that learning to read and understand the primary immunology literature is one of the most important and useful things they learn in the class. The app can be set to automatically back up work on a cloud service. Students tell us that they use Notability to read the papers because they can read them in color, enlarge figures to see them better, and easily mark up the papers and write comments. When explaining a point about a paper figure in class, it can be projected onto a screen from the iPad, and the instructor can mark up the figure in response to student questions. While many students use hard copies during the first paper discussion, we have found that, before the end of the class, most of the students are working from their iPads.

iPads are also useful for interactive group work, including concept maps, drawing of detailed pathways, and group presentations. Most students find that Notability works well for concept mapping and allows them to creatively link concepts. The maps, drawings, or other work done in the group can then be projected while the students present their work to the class.

iPads are superbly adapted for use in the lab, as lab manuals are easily accessed, and the results of experiments can be directly entered into the iPad (36) Students frequently use the camera to document results (observations on their mice, ELISA plate results, tissue culture contamination) which they can share with the instructor to ask questions or use in their final lab reports. Electronic lab notebooks are also improving in quality and increasing in use. Not without downsides, they do also offer many advantages, including providing the instructor with remote access to student data on a real-time basis.

When using iPads (or laptops) in the lab, students must have a prior safety briefing, for example, to ensure they don't bring laboratory contaminants home. When needed, protective sleeves, designed to protect iPads in the kitchen, can be provided for protection from powders and liquids. Additionally, we use iPads in the lab to discuss how to analyze and graph data. After students do their first ELISAs, they graph their results and upload them to the class Google Drive or the Learning Management System (Blackboard, Moodle, etc.) as pdfs. As the graphs are projected, the instructor can ask the class for feedback and write comments and suggested revisions directly on the graph or figure. This results in greatly improved figures.

One of the most important outcomes from using iPads in courses is that students become comfortable experimenting with different approaches to learning the material. This reinforces the point that acquiring information is an ongoing, constantly evolving process. The class winds up being a collaborative exercise between the students and the instructor throughout the term, as both experiment with new approaches, revising (or dropping) them as they go.

The cost of iPads is a critical consideration when in-class iPad use is being mandated. While a typical iPad costs less than most smartphones or laptops, their purchase price is nonetheless non-trivial for a college student, especially given the relatively short half-life of the device. When we first began providing iPads for students enrolled in the Immunology class, half of the students later purchased iPads for academic use, and the percent of students obtaining iPads continues to rise. This raises a difficult issue; students who might benefit from having an iPad for their academic studies, but cannot afford them, can be further disadvantaged. This is an ongoing issue, with no easy solutions, although providing low-cost or subsidized rental equipment for students with need offers one potential solution.

Overall, iPads can greatly enhance the teaching of immunology and facilitate active learning approaches. However, some faculty are not as comfortable with these devices as their students. Given the many ways to teach (and learn) immunology, iPads should be viewed as one of many exciting options in the toolbox to assist students on the challenging journey of learning about the immune system.

## Discussion

Teaching immunology to undergraduates offers unique rewards and challenges. In terms of rewards, the subject matter is engaging and easily connected to everyday life. Since the topic is usually offered as an elective, it tends to draw students with a keen interest and motivation to learn. Discussions of the immune response naturally lend themselves to review of basic areas of biological understanding and can help students to hone their facility with these areas of study. Likewise, connections of the discipline to medical, ethical and social issues are endless. Working with undergraduates offers an unparalleled opportunity to tap into the wonder that students at this level experience the first time they are faced with the beauty and complexity of the immune system. Their thinking is flexible, and because everything is new, nothing feels out of the ordinary. We have watched undergraduates effortlessly absorb concepts that we ourselves found difficult, simply because they lacked knowledge of earlier, engrained paradigms or preconceived notions of how things “should” work.

One of the main challenges we face as teachers is the diversity of scientific backgrounds that students bring to the course, which is highly dependent on prior coursework and experiential learning opportunities. Even when they have taken the foundational courses, concepts and terminology from associated fields are still quite new and therefore easily confused. It can be hard to know where to start with teaching immunology, and the mountain of new terminology does not help with this. Therefore, faculty must resist the temptation to try to cram too much into the course without attention to what the students need at this stage and are able to appreciate.

In this article, we have described a number of student-centered, active learning strategies that we have employed in our classrooms to enhance student motivation, comprehension and retention. Our hypothesis is that these strategies, which are time-consuming and take real effort to implement, enhance student comprehension and retention to a degree that makes the extra time and effort on the instructor's part demonstrably worthwhile. We are not aware of studies by teachers of immunology that compare student outcomes following courses that engage in active vs. passive learning. The “data” that we do have to share derives from student course evaluations and from many years of talking with alumni/ae regarding their perception of how their experiences in our courses prepared them for their future careers. Those of us who have taught for many years have seen a change in the ways in which our students best learn, and have striven to adapt our own teaching styles to the needs of our students. In a recent course, students were asked: “Tell me how useful (or not) you found the active learning exercises and which ones, if any, you found particularly useful.” Ninety percent of the students in this class found one or more of the exercises useful or very useful and a frequent comment in student responses was that the opportunity to take a moment in class to talk in a small group about difficult questions that were addressed by the learning exercise was helpful. As teachers, we have all seen how students who are too shy or intimidated to talk aloud within the class as a whole can come alive when small group exercises are offered. Notably, the few students who failed to find the exercises useful were either indifferent or left the answer blank, indicating that they did not feel that the exercises were a poor idea.

Two common themes arise in our collective teaching experiences; a need to prepare students before they enter the classroom and the desire to help students *do* rather than merely *view* immunology. These principles are at the core of our current understanding of best practices in undergraduate STEM teaching, and therefore hold true in any classroom setting.

We have by no means attempted to be comprehensive in this review, and we are certain that many other excellent examples of active learning applied to the immunology classroom exist. Two of the most common and successful active learning approaches, class discussions of the primary literature and laboratory experiences, are topics of other papers in this issue (10–12). A third active learning approach, Just-in-Time-Teaching (JiTT), is only briefly addressed here but covered in more depth in other papers in this issue (15).

These ideas are offered as one might present a smorgasbord; no one teacher can use all of these strategies in any one class, and the selection of activities must be carefully matched to the subtopic and to the student population. A lively group of sophomores may learn best if they are encouraged to move around the classroom, whereas a smaller group of graduate-school bound seniors might benefit more from group writing assignments. The experienced teacher knows the student population and their challenges, and will adjust accordingly.

Importantly, as we apply these ideas to the classroom, it is worth considering current student populations and present-day issues. Active learning approaches and mixed assessment methods have been shown to reduce achievement gaps, increase retention, and improve comprehension for all students, but especially in groups currently underrepresented in the sciences (4, 13, 37). However, one size does not fit all, and some student-centered or active learning approaches may not work for certain students or in specific settings. For example, anxiety is an increasing problem among high school and college-aged students. A study by Cooper and colleagues (38) found that some active learning approaches in a large classroom (e.g., cold or random call) elevated anxiety levels in students, as compared to conventional, lecture-based approaches. Clicker questions and group work have similarly been found to have the potential to either increase or decrease anxiety, depending on how they were administered. Cooper and colleagues outline several valuable strategies for reducing anxiety while employing specific active learning methods (38). The addition of active learning, like any new pedagogical approach, requires thoughtful implementation and regular assessment if we hope to enhance student learning and level the playing field. In fact, if we want to make these changes in our classrooms, building trust among and between students and their teachers may be crucial first steps (39).

In closing, this article presents implementation of a few pedagogical advances made in the last several decades, applied to the teaching of undergraduate immunology. However, we recognize that the field of STEM education is currently moving as quickly as the science it seeks to communicate, and we eagerly await new breakthroughs. As immunologists, we look forward to the opportunity to apply best practices from these rigorously evaluated methods to “infect” the next generation of undergraduates with the joy of learning about the immune system.

## Author Contributions

RP: conceived and organized the project. JO and SS: wrote the first drafts of the abstract, introduction, and discussion. All authors wrote sections of the manuscript and edited others, and focusing on the approaches used in their classrooms. SS: JiTT. FM and RP: video and audio supplements. FM and RP: clicker questions. SS: simulations, reenactments, and interactive in-class activities. JO, RP, and SS: in-class short presentations. FM, JO, and RP: concept maps. FM, JO, RP, and SS: writing projects. RP: iPads. The authors all contributed to manuscript revision, read and approved the submitted version. This paper was a deeply collaborative effort by all authors.

## Conflict of Interest

The authors declare that the research was conducted in the absence of any commercial or financial relationships that could be construed as a potential conflict of interest.

## Footnotes

^1^For examples, see: https://www.cimr.cam.ac.uk/research/principal-investigators/principal-investigators-a-h/griffiths and https://www.vet.upenn.edu/research/research-laboratories/research-laboratory/hunter-laboratory.

^2^https://www.playfactile.com/

^3^https://www.gingerlabs.com/
